# Mental health history—a contributing factor for poorer outcomes in burn survivors

**DOI:** 10.1186/s41038-017-0106-8

**Published:** 2018-04-06

**Authors:** Frank Li, Danielle Coombs

**Affiliations:** Concord Repatriation General Hospital, Sydney, Australia

**Keywords:** Burns, Mental health history, Outcomes

## Abstract

**Background:**

A pre-morbid mental health history is common in patients with severe burn injuries. This creates challenges in providing rehabilitation. The aim of this study is to cross examine the possible impact of psychological co-morbidities on outcomes.

**Methods:**

A notes audit was carried out examining patients that were admitted to Concord Hospital Burns Unit in a 3-year period (2010–2012). Patients with total body surface area (TBSA) of 20% or greater and aged between 16 and 50 years were included. Subjects were divided into a mental health group and a control group. SPSS version 21 statistic program was used for analysis the data.

**Results:**

Data collected included length of stay, time to achieve independence, %TBSA, types of burns and surgery required. Results of 69 files showed that the average length of stay per %TBSA was nearly double in the patients with a mental health problem (1.47 vs 0.88). They also had a higher rate of re-graft (52% vs 22%) due to infection and poor nutrition. The average time for patients to achieve independence in daily living activity was significantly higher (*p* = 0.046) in the mental health group (36.2 days) versus the control group (24.1 days).

**Conclusion:**

Patients with a mental health history may have poorer general health. This may result in a higher failure rate of grafting, leading to a requirement of re-graft. Hence, it took a longer time to achieve independence, as well as a longer hospital stay. A mental health history in burn survivors can be a contributing factor for poorer outcomes in the adult population.

## Background

Burns can be one of the most devastating and life-changing injuries the human body can survive. Burns can have a large impact on the psychological, physical and social well-being of the survivors. Rehabilitation for this population can be a lifelong process. Psychological well-being can have a huge impact on the survivor and subsequently the outcomes of rehabilitation. In current literature, there are many factors that have shown to predict outcomes in burn injury. Predictors for mortality include age, total body surface area (TBSA) and inhalation injury [1]. Survivors who are young, married, employed and higher functioning demonstrate the best outcomes in burn rehabilitation [2].

A pre-morbid mental health history is common in patients with severe burns to the point [3–8]. Self-harm or self-inflicted burn injuries were certainly leading to financial impact to the society. There were many causes of intentional burn injury, and mental illness was common in severe burns [9, 10]. This can create challenges for therapists in providing rehabilitation to these patients. In fact, mental health was also associated with physical malformation [11]. Rehabilitation is an imperative part of the recovery process. Compliance and participation in therapy can determine functional outcomes. It is important for the survivors to practice self-management. Exercise and stretching regimes can be painful and very difficult. It is important that burn survivors are motivated to continue such long process of rehabilitation. Survivors need to learn to manage their wounds, medication, nutrition, scars, pressure garments, exercise and stretching programs on a day-to-day basis. The aim of this study is to evaluate the impact of a pre-morbid mental health history on physical outcomes following a severe burn injury.

## Methods

A retrospective medical notes review was carried out as a clinical quality improvement to examine burn patients admitted to Concord Hospital Burns Unit during the period from January 2010 to December 2012. The study was approved by the Ethic Committee of the Sydney Local Area Health District. Patients with a TBSA of 20% or greater and aged between 16 and 50 years were included. Patients older than 55 years were excluded due to the possible risk of co-morbidities negatively affecting the outcomes. Concord Hospital Burns Unit covers a large area of the state of New South Wales. Hence, many patients transferred from various regional hospitals and facilitates. Patients with official diagnosis with mental health history by psychiatrists were identified in the mental health group. Those burned patients with mental health history were diagnosed by psychiatrists in other institutions prior to their burn incidents. According to the medical notes, these patients had regular reviews by the local psychiatrists and psychologists. Their conditions were controlled by medications as well as psychology intervention. Data would not include for those patients being diagnosed during the hospitalisation at the Burns Unit from post-traumatic stress symptom.

Evidence shows that recovery from serious injury and subsequent outcomes are poorer in the older adult population [12], and thus, it was deduced that this population had a greater incidence of co-morbidities that affect burn outcomes. Other co-morbidities were not collected in the data. Information such as age, gender, mechanism of burn, area of burn, and mental health history was recorded. Data collected included hospital length of stay, whether a re-graft was required, reason for re-graft, time taken, time for the first mobilisation, time taken to achieve independence with mobility, time taken to achieve independence with activities of daily living (ADL) and the discharge location.

SPSS version 21 statistics program was used for the analysis. Results were analysed by comparing the mental health group with the control group using the non-parametric test (Mann-Whitney *U* test). *p* < 0.05 is being considered as the significant level.

## Results

There were 69 appropriate participants; they were divided into the mental health group (*n* = 24) and the control group (*n* = 45). Mean age of the mental health group was 35.4 years, and mean age of the control group was 30.6 years. Mean TBSA of the mental health group was 31.06%, and mean TBSA of the control group was 30.89%. 50% of the identified patients with mental history were being diagnosed with schizophrenia. The remaining 26.4% were drug-induced psychosis and 23.6% were diagnosed with personality disorder and depression.

Demographic data and results of statistical analysis of the two groups are listed in Table 1. Although it did not reach a high significance level, the average age of the mental health group was slightly higher than the normal group. Within the eligible list, the TBSA was not significantly different between the two groups. There were 15 (62.5%) male patients being identified with mental health issues. The majority of patients were being discharged home, and only 2 patients in the mental health history group were discharged to mental health facility. Amongst the other patients being labelled with mental health issues, 6 patients were referred to other rehabilitation services and 2 were discharged to their local hospitals rather than home. However, only 1 patient in the control group was being discharged to another rehabilitation service. Thus, 97.8% of patients in the control group was being discharged back home versus only 56.5% of the mental health group patients were being discharged back home.

**Table 1:** Demographic data and results of statistical analysis of the two groups

Variables	Mental health group	Control group	Significance level
Gender			
Male	15	39	
Female	9	6	
Age (mean, SD)	35.42 (10.38)	30.60 (9.78)	0.067
TBSA (mean, SD)	31.06 (9.66)	30.89 (11.48)	0.578
Length of stay (mean, SD)	45.83 (33.26)	27.24 (21.88)	0.001**
Stay per %TBSA (mean, SD)	1.47 (1.00)	0.88 (0.44)	0.001**
Time first mobilise			0.131
25th percentile	3	2	
Median	5	4	
75th percentile	11	7	
Days indep. mobilise			0.097
25th percentile	8	10	
Median	19	14	
75th percentile	31	19.5	
Days indep. ADL			0.035*
25th percentile	20.5	15.25	
Median	25.0	20.0	
75th percentile	43.5	27.5	
No. of graft (mean, SD)	0.83 (0.38)	0.80 (0.41)	0.738
No. of re-graft (mean, SD)	0.55 (0.51)	0.25 (0.44)	0.021*

*p <0.05 and **p <0.01

*TBSA* total body surface area, *SD* standard deviation, *ADL* activities of daily living

Severe burned patients with a mental health history had an average hospital length of stay of 45.8 days (standard deviation (SD) of 33.3) and the median of 39 days and the calculated stay per %TBSA of 1.47 days. Control group had a length of stay of 27.2 days (SD of 21.9) and the median of 24 days and the calculated stay per %TBSA of 0.88 days. Hence, there was a significant lower length of stay for patients without mental health issues (*p* = 0.001).

Variables such as time to first mobilisation, days achieved independent mobilisation and days achieved independent ADL demonstrated a high variation. Thus, non-parametric test was used for the analysis (Table 1). Time to first mobilisation was earlier in the control group, but it did not reach significant level. Similarly, the control group was able to achieve independent mobilisation quicker than the mental health group. Time to achieve independence with mobility was not significantly higher in the mental health group when compared to the control (*p* = 0.097).

The average time for patients to achieve independence was 28.1 days (SD of 23.5 and the median of 22 days). It was significantly (*p* = 0.035) higher in the mental health group (36.2 days with standard deviation of 28.8) versus the control group (24.1 days with SD of 19.4).

The requirement for grafting was similar between the two groups, and there was no significance being found (*p* = 0.74). However, there was a significantly (*p* = 0.02) higher rate of re-graft due to infection and poor nutrition in the mental health group when compared with the control (52% vs 22%).

## Discussion

Pre-morbid mental health co-morbidities are common amongst burn survivors. The data suggests that the presence of mental health history can negatively impact outcomes. Factors such as compliance, general health and social situation may contribute to this. The success of burn rehabilitation process depends on the willingness of patients doing their self-management program. Even considered as in-patient rehabilitation program, the interacting time of therapist with individual patient would be less than 1 h per day. Taking away 8 h of sleep and 4 h of attending personal hygiene, there are still 12 h free times. In addition to the formal rehabilitation program, patients are often requested to exercise every hour of self-stretching and exercise. Compliance with therapy and self-management is likely to be lower in the mental health patient group which may account for a longer time to achieve independence with ADL.

Infection and poor nutrition are being the major factors affecting wound healing. Many burned patients are recommended to have additional protein intake to facilitate wound healing. There is a risk of excessive nutrition being converted to fat, so burned patients are recommended to have a corresponding exercise program. Personal hygiene is essential to avoid wound infection. The presence of germs in the wound would destroy the graft; hence, the healing time would be much delayed. Due to the delayed healing, it can result in further mental and physical suffering as well as scarring. The authors found that the rate of re-graft due to infection and poor nutrition was significantly higher in the mental health group when compared to the control. This could suggest poor general health and subsequent ability to fight infections as well as the possibility of poor compliance with a healthy lifestyle, which included inappropriate oral intake amongst patients with mental health history.

Many burned patients are discharged from Burns Centers in developed countries prior to complete wound healing. This depends on their abilities to return to the outpatient dressing clinic and to carry out self-management. Quite often, patients with mental health issues were being kept in the hospital for a longer period of time knowing that they were not able to self-care for the wound even though it was minor. This would increase the cost of health care for this particular group of patients. In general, 1 day per %TBSA is expected being the benchmark target of the Burns Unit at Concord Hospital. Patients in the control group were able to meet the target, but not for patients with mental health history. A combination of the above reasons may lead to increased length of stay per %TBSA and thus increased economic burden.

In order to provide proper care to this particular group of patients, special considerations are needed. Identifying the potential for poorer outcome in this patient population is important in burn management and rehabilitation. Clinical implications include the implementation of personal exercise programs, behaviour support and compliance plans, prompt referrals to mental health services and combined therapy sessions with other therapists, psychologists and diversional therapists. Further research into the impact of the implementations of these techniques is required.

The nature of this notes audit as a clinical quality improvement activity leads to limitations. The exclusion criteria for patient selection were only age and TBSA. Co-morbidities of the younger population were not collected and taken into account and thus may have had an impact on the results. Further research into the relationship between mental health history and rehabilitation outcomes in burn survivors is required.

## Conclusion

Severe burn survivors with a mental health history are more likely to have poorer outcomes in rehabilitation. Length of stay, time to achieve independence with mobility, time to achieve independence with ADL and rate of re-graft showed to be increased in the mental health group when compared to the control. Factors such as compliance, general health and psychological status may account for these differences. This study highlights the importance of recognising the potential for poorer outcomes and thus implementing individualised programs to encourage compliance in this patient group to reduce morbidity in burn survivors.
